# SUMO and the DNA damage response

**DOI:** 10.1042/BST20230862

**Published:** 2024-04-17

**Authors:** Jai S. Bhachoo, Alexander J. Garvin

**Affiliations:** SUMO Biology Lab, School of Molecular and Cellular Biology, Faculty of Biological Sciences, University of Leeds, Leeds, West Yorkshire LS2 9JT, U.K.

**Keywords:** DNA damage response, DNA synthesis and repair, genome integrity, post translational modification, SUMOylation, ubiquitin signalling

## Abstract

The preservation of genome integrity requires specialised DNA damage repair (DDR) signalling pathways to respond to each type of DNA damage. A key feature of DDR is the integration of numerous post-translational modification signals with DNA repair factors. These modifications influence DDR factor recruitment to damaged DNA, activity, protein-protein interactions, and ultimately eviction to enable access for subsequent repair factors or termination of DDR signalling. SUMO1-3 (small ubiquitin-like modifier 1-3) conjugation has gained much recent attention. The SUMO-modified proteome is enriched with DNA repair factors. Here we provide a snapshot of our current understanding of how SUMO signalling impacts the major DNA repair pathways in mammalian cells. We highlight repeating themes of SUMO signalling used throughout DNA repair pathways including the assembly of protein complexes, competition with ubiquitin to promote DDR factor stability and ubiquitin-dependent degradation or extraction of SUMOylated DDR factors. As SUMO ‘addiction’ in cancer cells is protective to genomic integrity, targeting components of the SUMO machinery to potentiate DNA damaging therapy or exacerbate existing DNA repair defects is a promising area of study.

## Introduction to SUMOylation

SUMOylation is a protein post-translational modification whereby SUMOs are covalently attached to target lysine residues. Mature SUMO2 and SUMO3, share 97% amino acid identity and are referred to as SUMO2/3. SUMO1 is more distantly related to SUMO2/3 (∼47% amino acid identity) [1]. SUMO4 is not processed into a mature form so it is not conjugated [2,3]. SUMO5/SUMO1P1 regulates PML-NB (promyelocytic leukaemia (PML) nuclear bodies) but is designated as a pseudogene [4].

The maturation of SUMO precursors by SUMO proteases (SENP1 and SENP2) initiates the SUMOylation cycle by exposing C terminal di-glycine residues on SUMOs. The E1 heterodimer (SAE1:SAE2) charges the exposed di-glycine residue, and an E2 conjugating enzyme (UBE2I/Ubc9) catalyses the transfer of SUMO from E1 to the target protein. SUMO E3 ligases assist E2s in target recognition and formation of the isopeptide bond linking SUMO1-3 glycine residues and substrate lysines. SUMO E3s include PIAS1-4 (protein inhibitor of activated STAT 1-4), ZNF451, NSMCE2 (NSE2 (MMS21) homolog, SMC5-SMC6 complex SUMO ligase), RANBP2 (RAN binding protein 2) and other factors with E3-like properties [1]. SUMOs are conjugated as monomers, multi-monomers, and polymers. SUMOylated lysines are most abundant within ψKxE/D-type motifs (ψ = hydrophobic amino acid) although many SUMOylation sites are non-consensus [5].

SUMO1-3 are deconjugated by SENP proteases (SENP1-3 and 5-7) [6,7], USPL1 (ubiquitin specific peptidase-like 1) [8,9] and DeSi1/2 (DeSUMOylating isopeptidases 1/2) [10]. SENP1/2 deconjugate SUMO1. All SENPs deconjugate SUMO2/3, and SENP6-7 are specialised for poly2/3SUMO deconjugation [11,12].

To regulate cellular processes, SUMO facilitates protein-protein interactions with SUMO binding domains (SBDs). The most characterised are SUMO interaction motifs (SIMs or Type I). SIM consensus requires three or four hydrophobic residues flanked by negatively charged or phosphorylated residues [13]. Type II interactors bind the E67 loop of SUMO1 opposite the SIM binding groove used by Type I interactors [14,15]. Two further SBDs have been identified: the MYM zinc finger [16] and the ZZ domain (Type III interactors) [17]. Furthermore, yet uncharacterised, SBDs can be inferred from SUMO interactions not involving these domains/motifs [18]. SUMOylation and ubiquitination co-operate in DNA damage repair (DDR) signalling [19,20]. SUMO-targeted ubiquitin ligases RNF4 and RNF111 (STUbL) are ubiquitin ligases that recognise SUMOylated proteins through SIMs, promoting ubiquitin dependent proteasomal degradation and generating mixed SUMO-Ub conjugates [11,12,21].

Given its critical role, disruption in SUMOylation lead to cancers, cardiac disease, neurodegeneration, and inflammatory disease [22]. SUMOylation also has roles in organism development, spanning germ cells to tissue and organs [23].

Here we provide an overview of each of the main DDR pathways in which SUMO signalling has been investigated.

## Base excision repair and single strand break repair

**Figure 1.**
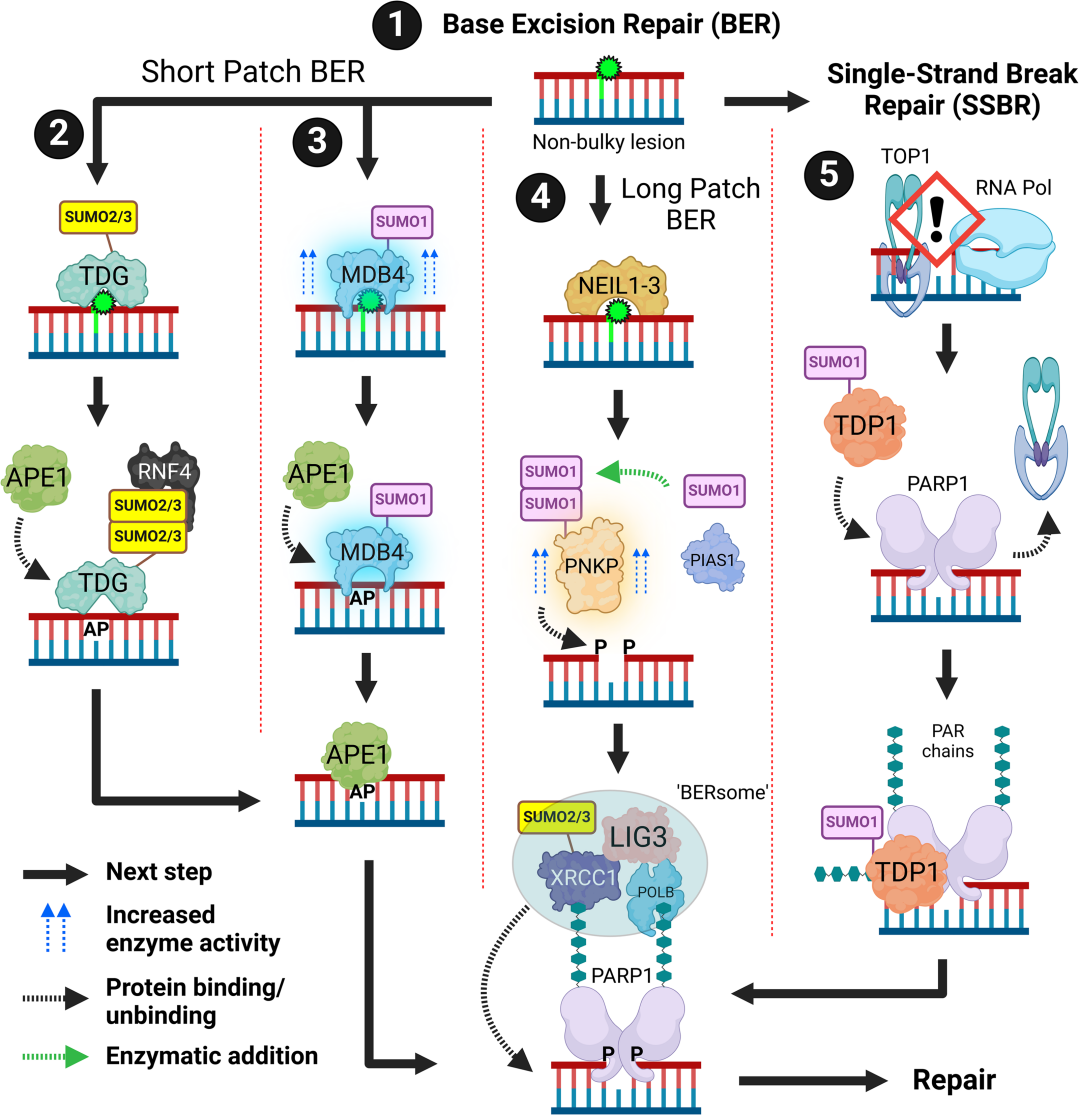
Base excision repair (BER) and single strand break repair (SSBR). (**1**) BER is initiated in response to non-bulky DNA lesions (green DNA base). (**2**) TDG recognises non-bulky lesions. TDG SUMOylation promotes interaction with RNF4. TDG SUMOylation also promotes APE1 binding to AP sites. APE1 then interacts with PARP1 and ‘BERsome’ associated proteins. (**3**) MDB4 also recognises non-bulky lesions. MDB4 SUMO1ylation increases its thymine glycosylation activity *in vitro*. This results in an apurinic/apyrimidinic (AP) site. APE1 recognises the AP site and binds leading to DNA repair via PARP1 and ‘BERsome’ associated proteins. (**4**) In the long patch pathway NEIL1-3 and PNKP are used, PNKP is SUMOylated which increases its phosphatase activity. XRCC1 SUMOylation promotes the recruitment of the ‘BERsome’ which contains the required nucleases for repair. Recruitment of the ‘BERsome’ is thought to be promoted by PAR chains. (**5**) SSBR can be considered a sub-pathway of BER. TOP1-induced SSBs are formed by collisions with an elongating RNA polymerase. PARP1 binds to ssDNA breaks which initiates autoPARylation of PARP1. SUMOylated TDP1 binds to C-terminus of PARP1 resulting in improved SSBR. Created with BioRender.com.

DNA bases are altered through oxidation and alkylation which is reversed by base excision repair (BER) (Figure 1). BER can be divided into two sub-pathways. Short-patch BER is initiated by DNA glycosylases which cleave the bond between the base and deoxyribose sugar, leaving apurinic/apyrimidinic (AP) sites (Figure 1, subsections 2 and 3). *In vitro*, SUMOylation of TDG (thymine DNA glycosylase) promotes dissociation from AP sites [24,25]. The significance of this to TDG's cellular BER function is not clear. AP sites are recognised by APE1 (AP endonuclease 1) which cleaves the DNA backbone. SUMOylation of TDG promotes interaction with APE1 and RNF4. RNF4's SUMO binding and ubiquitin ligase activity are required for DNA demethylation. RNF4 deficiency causes G:T mismatch repair defects [26]. The G:T/U mismatch glycosylase MBD4 (methyl-CpG binding domain 4) is SUMOylated in response to DNA damage. *In vitro,* SUMO1-modified MBD4 has higher thymine glycosylase activity [27]. The scaffold protein XRCC1 (X-ray repair cross complementing 1) recruits BER repair factors including POLB (polymerase β), LIG3 (ligase 3) and PARP1 (poly (ADP-ribose) polymerase 1), which together replace the correct nucleotide and seal the nick in the DNA backbone. XRCC1 is SUMOylated [26,28–30]. The interaction between the dual SUMO-ubiquitin E3 TOPORS (TOP1 binding arginine/serine rich protein) and XRCC1 is promoted by PARP1-generated PAR (poly-ADP-ribose) chains and is required for cellular resistance to the DNA methylating agent MMS (methyl methane sulfonate) [31]. SUMOylation of XRCC1 also promotes interaction with TDG via a SIM motif but does not affect the interaction with POLB and LIG3. *In vitro*, SUMOylated XRCC1 promotes the formation of a DNA-bound ‘BERsome’ which contains all the proteins needed for BER [30]. For long-patch BER, NEIL1-3 (Nei-like DNA glycosylase) generates single nucleotide gaps with a 3′phosphate end which is removed by the phosphatase PNKP (polynucleotide kinase 3′-phosphatase) (Figure 1, subsection 4). PNKP phosphatase activity is stimulated by PIAS1-dependent SUMOylation [32]. Single-strand break repair (SSBR) uses many BER components and, as such is considered a BER sub-pathway (Figure 1, subsection 5). Tyrosyl DNA phosphodiesterase 1 (TDP1) has multiple DNA repair functions, including SSBR. Treatment with the TOP1 (topoisomerase-1) poison camptothecin (CPT) promotes collisions between TOP1-DNA adducts and RNA polymerase II resulting in SSB formation. This results in the recruitment of TDP1 which is SUMO1ylated promoting transcription blocking SSB resolution [33,34]. PARP1 also aids in the repair of TOP1 adducts by PARylating and interacting with SUMOylated TDP1, promoting increased TDP1 stability and recruitment of XRCC1 which enables SSBR [34].

## Nucleotide excision repair

Nucleotide excision repair (NER) resolves bulky DNA adducts such as ultraviolet radiation (UVC)-induced cyclobutane pyrimidine dimers (CPDs) and 6,4 photoproducts (6-4PP). Global-genome NER (GG-NER) recognises lesions in transcribed and non-transcribed DNA via sensor proteins DDB1-DDB2 (damage specific DNA binding protein 1/2). Transcription-coupled NER (TC-NER) uses the distortion recognition feature of RNA-polymerase-II complexes to signal for repair during transcription, via CSA-CSB (Cockayne syndrome WD repeat protein CSA/B).

**Figure 2.**
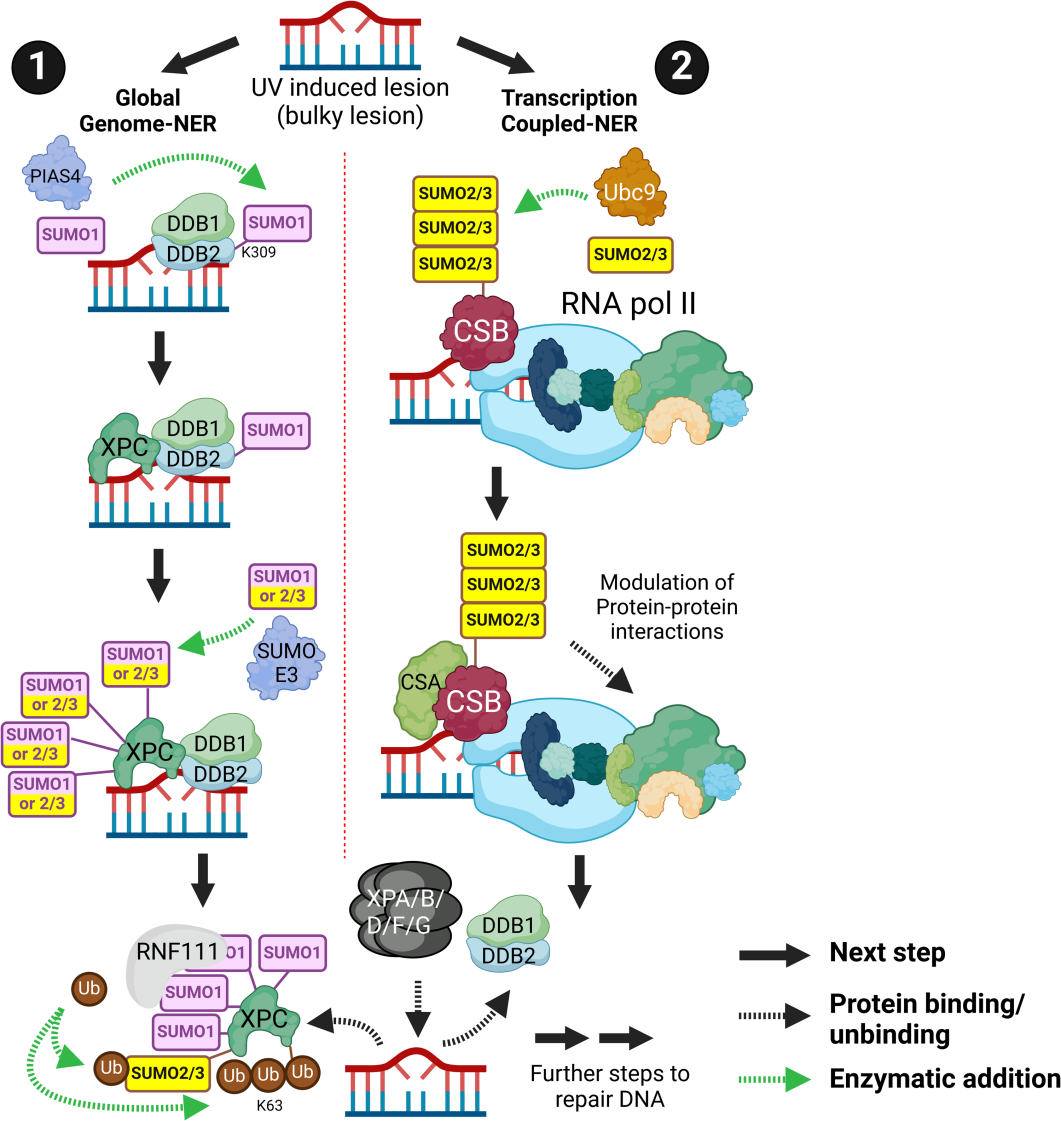
Nucleotide excision repair (NER). NER is initiated in response to bulky DNA lesions (**1**) Global-genome NER requires SUMOylation. DDB2 is SUMO1ylated leading to the recruitment of XPC. XPC can be SUMO1ylated and/or SUMO2/3ylated at multiple potential residues. SUMOylation allows RNF111 recruitment leading to non-degradative K63-Ub chain formation on XPC and facilitates its removal. Subsequent NER factors are recruited to complete repair. DDB1/DDB2 is also removed, and DNA is eventually repaired. (**2**) SUMOylation is required in Transcription-coupled NER. CSB is associated with the lesion and is SUMO2/3ylated by Ubc9. CSA binds to SUMOylated CSB and its interactome including components of RNA-Pol II is altered. XPA/B/D/F/G is recruited to the lesion and leads to repair. Created with BioRender.com.

UVC promotes SUMOylation of DNA-bound DDB2 (Figure 2, subsection 1). This facilitates subsequent XPC recruitment and clearance of CPDs [35,36]. Transcription-blocking DNA lesions induce the SUMOylation of CSB. SUMOylation defective CSB is less able to recruit to UVC-damaged DNA and has altered interactions with components of the RNA-POL2 complex (Figure 2, subsection 2). This results in delayed post-UVC transcriptional recovery [37,38]. XPC aids the assembly of subsequent NER factors XPA/B/D/F/G (xeroderma pigmentosum group A/B/D/F/G-complementing protein). XPC SUMOylation is enhanced by UVC [38–41]. SUMOylation of XPC promotes clearance from damaged DNA via RNF111 which generates non-degradative SUMO dependent ubiquitin K63 linkages. Cells expressing SUMOylation defective XPC are deficient in CPD and 6-4PP processing, clear slowly from CPD lesions and fail to permit proper recruitment of XPA, B, F and G [42,43]. These factors are required to incise the DNA to allow repair synthesis and sealing of nicked DNA. Therefore, SUMOylation of XPC acts as a clearance signal, enabling the loading of downstream incision factors to access DNA.

## Non-homologous end joining

Non-homologous end joining (NHEJ) is the dominant DSB (double-strand break) repair pathway and occurs predominantly in G1 and to a lesser extent throughout the cell cycle. During NHEJ, Ku70/Ku80 heterodimers bind and protect the ends of broken DNA and recruit the nucleases and polymerases required to process and ligate the ends together. The re-ligation may result in the loss of genetic material so can be mutagenic. Unlike the other major DSB repair pathway HR (homologous recombination) NHEJ does not require extensive end-resection of DNA surrounding a DSB.

**Figure 3.**
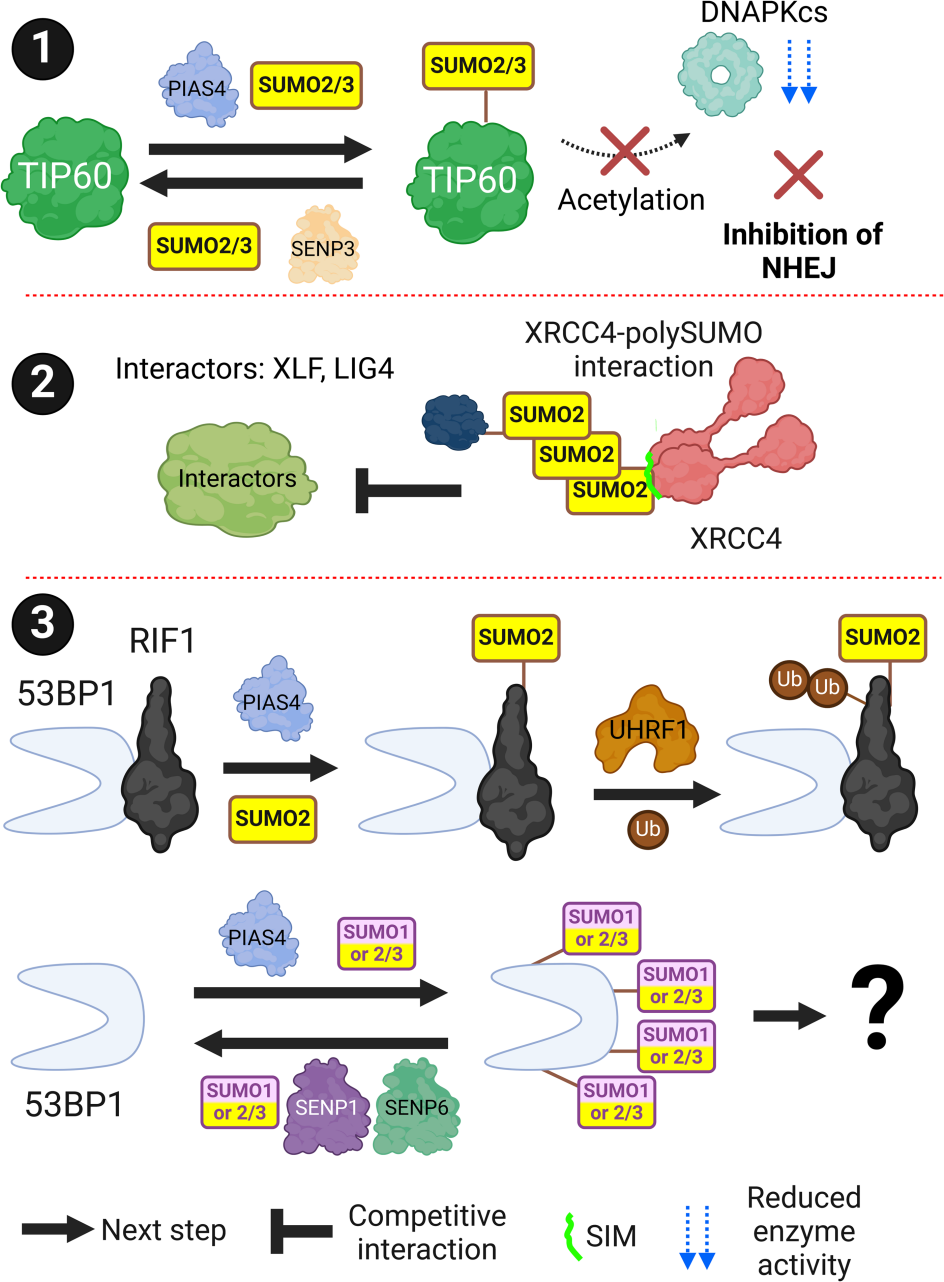
Non-homologous end joining (NHEJ). SUMOylation is required at multiple stages of NHEJ. (**1**) SUMO2/3ylation of acetyltransferase TIP60 by PIAS4 results in reduced interaction with DNAPKcs and acetylation. This reduces DNAPKcs autophosphorylation and NHEJ signalling. (**2**) XRCC4 can interact with polySUMO2 chains. These interactions are mutually exclusive with some XRCC4 interactors. (**3**) 53BP1 interactor RIF1 can be SUMO2ylated by PIAS4 at multiple residues. This leads to UHRF1-dependent ubiquitination and RIF1 removal from 53BP1-associated DSBs. 53BP1 can also be SUMOylated at multiple residues but its effect is not known. Created with BioRender.com.

DNAPKcs (DNA-dependent protein kinase, catalytic subunit) is a kinase that becomes active on binding to Ku70/Ku80. DNAPKcs phosphorylates multiple DNA repair and checkpoint proteins. TIP60 acetylates DNAPKcs promoting its autophosphorylation and activation. During S-Phase TIP60 is SUMOylated by PIAS4 which reduces its interaction with DNAPKcs and acetylation to limit NHEJ [44] (Figure 3, subsection 1). XRCC4 (X-ray repair cross complementing 4) recruits several NHEJ repair factors including XLF (XRCC4-like factor) and LIG4 (ligase 4). XRCC4 is a SUMO2 chain receptor, which modulates its ability to interact with various NHEJ subcomplexes [18,44]. XRCC4 is also SUMOylated which regulates its nuclear localisation and NHEJ repair activity [45] (Figure 3, subsection 2).

TP53BP1 (tumour protein P53 binding protein 1/53BP1) is a pro-NHEJ chromatin-binding protein that limits DNA end-resection through competition with BRCA1-BARD1. 53BP1 recruits the RIF1 (Rap1-interacting factor 1)/Shieldin/CST (CTC1-STN1-TEN1) complexes which have roles in limiting DNA end resection and Polymerase-α primase-dependent gap filling [46]. 53BP1 is SUMOylated on DSB induction by SUMO1 and SUMO2/3 and deSUMOylated by SENP1 and SENP6 ([47–53] (Figure 3, subsection 3). Despite abundant SUMOylation of 53BP1, the direct role SUMOylation has on 53BP1 function is unknown. RIF1 (Rap1-interacting factor 1) is also SUMOylated in response to DNA damage which aids in UHRF1 (ubiquitin like with PHD and ring finger domains 1)-dependent ubiquitination and removal from sites of damage (Figure 3, subsection 3) [51,54].

## Homologous recombination

HR is a less error-prone pathway restricted to S/G2-phases of the cell cycle, as it uses the sister chromatid as a homology template for repair. HR is the most intensely studied DDR pathway in the mammalian SUMO signalling field [55], with SUMOylation having important roles in DNA end-resection, DSB-chromatin signalling and RAD51 filament formation.

## DNA end resection

**Figure 4.**
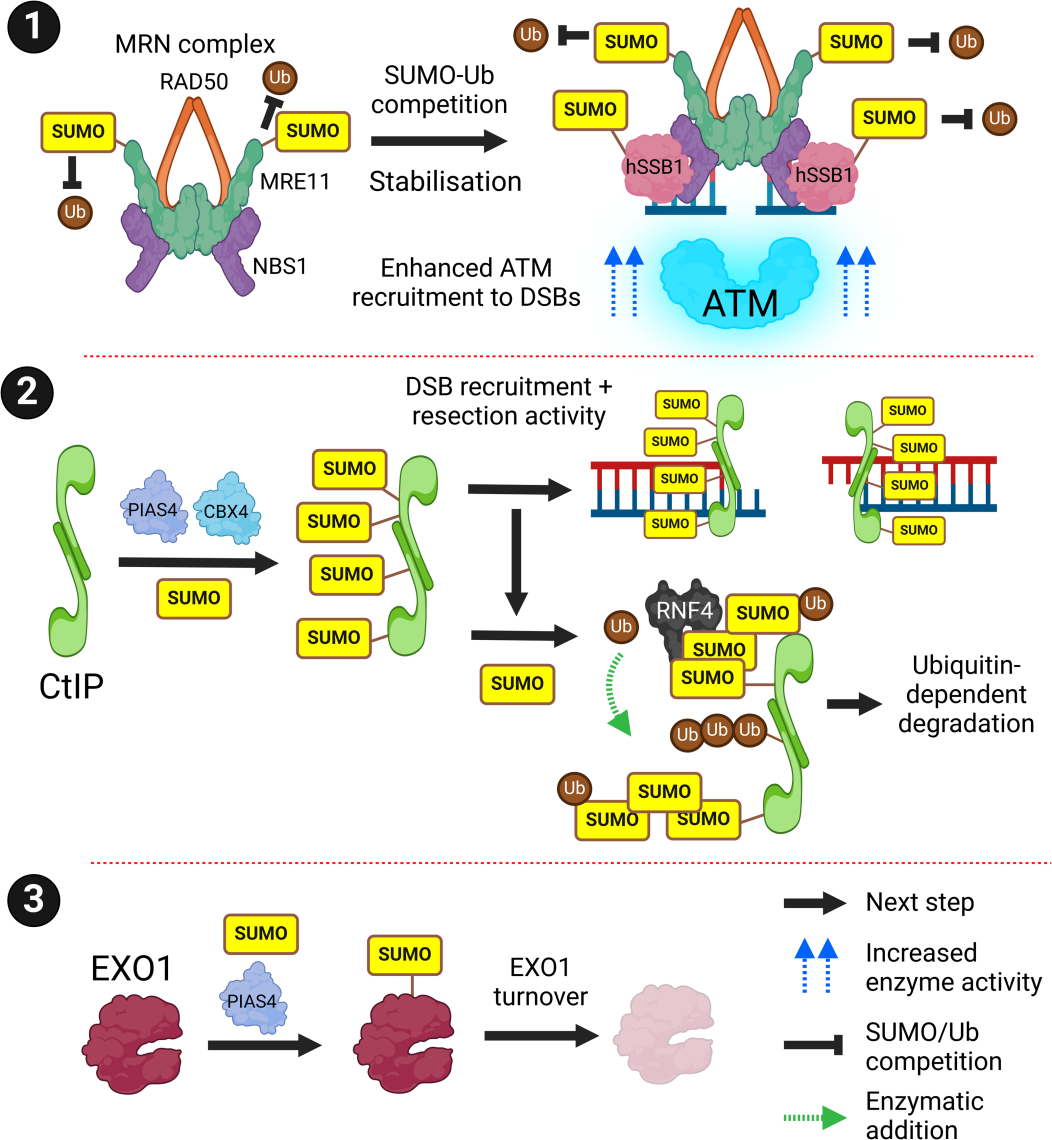
SUMO and DNA end resection. During early HR, the MRN complex is recruited to DSBs. (**1**) MRE11 of the MRN complex and hSSB1 are SUMOylated which competes with ubiquitination and enhances ATM recruitment and activity at DSBs. (**2**) The MRN interactor CtIP is SUMOylated by PIAS4 and CBX4. This promotes its recruitment to DSBs for subsequent short-range DNA end resection. However, CtIP can be hyperSUMOylated to form SUMO chains, resulting in RNF4-dependent ubiquitination and degradation. (**3**) EXO1, which is responsible for long-range DNA end resection is SUMOylated by PIAS4 and deSUMOylated by SENP6 which regulates its stability by an unknown mechanism. Created with BioRender.com.

HR requires carefully controlled DNA end-resection to generate single-stranded DNA. Several nucleases are involved in different steps of the HR pathway. Treatment with SUMOylation inhibitors, or interference with components of the SUMO system results in significant end-resection defects [56–58]. MRE11 is the endo/exonuclease component of the MRN (MRE11A-RAD50-NBS1) complex which is one of the first proteins recruited to DSBs where it performs the initial steps of DNA end-resection in addition to recruitment of the DSB master kinase ATM (ataxia telangiectasia mutated) (Figure 4, subsection 1). MRE11A is SUMOylated following DSB generation [44,59–61]. SUMOylation stabilises MRE11A by preventing its ubiquitination and degradation [61]. SUMOylation also stabilises the NBS1 interactor hSSB1 (single-stranded DNA-binding protein 1) enhancing ATM recruitment to DSBs [62].

The MRN interactor and endonuclease CtIP (CTBP-interacting protein) perform the short-range DNA end resection steps and undergo SUMOylation in response to DNA damage (Figure 4, subsection 2) [28,29,49,50,52,56–58]. SUMOylation of CtIP supports its recruitment to DSBs, DNA-resection and subsequent RAD51 loading [56–58]. CtIP is SUMOylated by two DSB localised SUMO E3s, PIAS4 and CBX4 [48,56–58,63]. CtIP SUMOylation promotes its RNF4-dependent ubiquitination and degradation, [50,57]. EXO1 (exonuclease 1) promotes the second wave of long-range DNA end-resection and is also SUMOylated by PIAS4 and deSUMOylated by SENP6, which regulates its stability independently of RNF4 (Figure 4, subsection 3). Thus, SUMOylation has an important role in regulating the amplitude and termination of DNA end-resection through regulated protein turnover [64].

## DSB-chromatin signalling

**Figure 5.**
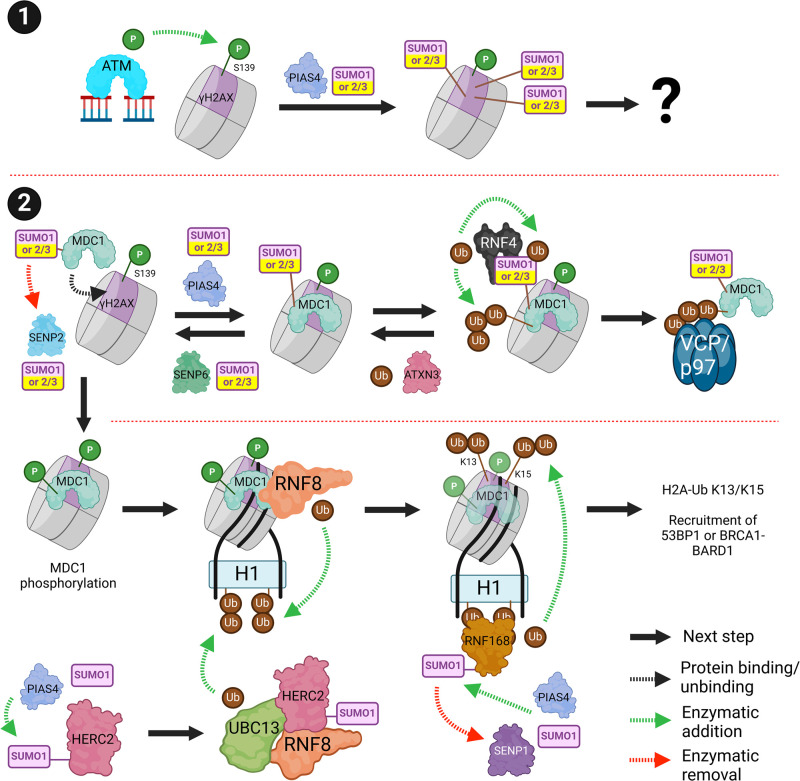
DSB-chromatin signalling. SUMOylation is critical for DSB signalling at chromatin. (**1**) When ATM is recruited to a DSB, it phosphorylates H2AX (γH2AX). γH2AX is SUMOylated by PIAS4 but the effect of this is unknown. (**2**) γH2AX recruits MDC1. MDC1 is SUMOylated by PIAS4 and deSUMOylated by SENP2/6. SUMOylated MDC1 can be removed by RNF4-dependent ubiquitination and VCP/p97-dependent clearance. RNF4-dependent ubiquitination is opposed by ATXN3 to prevent premature removal of MDC1. MDC1 is phosphorylated and recruits RNF8 which ubiquitinates histone H1. RNF168 recruits to the ubiquitinated H1 which then ubiquitinates γH2AX at K13/15. γH2AX ubiquitination leads to 53BP1 (NHEJ) or BRCA1-BARD1 (HR) recruitment. RNF168 is SUMO1ylated by PIAS4 and deSUMOylated by SENP1 to regulate its spreading on chromatin. Created with BioRender.com.

Recruitment of ATM to DSBs results in phosphorylation of histone H2AX at Ser139 (γH2AX). γH2AX is SUMO1ylated at multiple residues by PIAS4, although the function of γH2AX SUMO1ylation is currently unknown [66] (Figure 5, subsection 1). MDC1 (mediator of DNA damage checkpoint 1) is recruited to γH2AX [65] where it is SUMOylated by PIAS4 which promotes recognition by RNF4, ubiquitination and VCP/p97 (Valosin-containing protein) dependent extraction from DSBs [66–70]. The basal SUMOylation state of MDC1 is regulated by SENP2, which dissociates from MDC1 after DSB induction. Loss of SENP2 causes premature RNF4-VCP-dependent eviction of MDC1 from DSBs in G1, and failure in downstream ubiquitin signalling [70]. Loss of RNF4 causes retention of MDC1 at DSBs which is harmful to the DSB response [66–70]. The DUB (de-ubiquitinating enzyme) Ataxin-3 antagonises the RNF4-dependent ubiquitination of MDC1 maintaining its retention at DSBs [71,72]. As many SUMO-E3s are autoSUMOylated and recruited directly to DSBs [48,63], DSB-localised RNF4 has an additional regulatory role in limiting or terminating DSB-associated SUMOylation by promoting SUMO-E3 ubiquitination and turnover [51]. SENPs also play an important role in regulating SUMO signal dynamics by protecting substrates from excessive RNF4-driven degradation, coordinating the timing of recruitment to damaged DNA, and preventing SUMO-SIM accumulation in nuclear condensates [20,47,70,73,74].

Phosphorylated MDC1 recruits RNF8 which ubiquitinates histone H1 [75]. PIAS4-dependent SUMOylation of HERC2 (HECT and RLD domain containing E3 ubiquitin protein ligase 2) facilitates its interaction with RNF8-Ubc13 and promotes ubiquitination at DSBs [76]. RNF168 interacts with ubiquitinated H1 [75] and in turn, ubiquitinates H2A/H2AX at K13/K15 which can recruit 53BP1 or BRCA1-BARD1 (breast cancer type 1 susceptibility protein — BRCA1 associated RING domain 1) [77–80]. PIAS4 SUMO1ylates and SENP1 deSUMOylates RNF168, which maintain correct 53BP1 foci expansion and limit NHEJ [81,82]. The PIAS4-dependent SUMO1 conjugation wave detected at DSBs [48,83] has been proposed to be composed of mixed SUMO1-SUMO2/3 linkages specifically generated by PIAS4 [29] (Figure 5, subsection 2).

## BRCA1-BARD1

BRCA1-BARD1 heterodimers are recruited to DSBs through multiple routes including RNF168-dependent ubiquitination of H2A/H2AX at K13/K15 [77–79]. BRCA1-BARD1 is an E3 ubiquitin ligase for H2A at K125/127/129 which promotes chromatin reorganisation and limits 53BP1 spreading [84,85]. BRCA1 also interacts with PALB2 (partner and localiser of BRCA2) which bridges interaction with the RAD51 loader BRCA2 (breast cancer type 2 susceptibility protein), and CtIP which performs DNA end resection. BRCA1-BARD1 are both SUMOylated during the DDR response [47,49,50,83]. SUMOylation of the N terminus of BRCA1 stimulates its ubiquitin E3 ligase activity [83]. Similarly, to 53BP1, upstream SUMOylation at DSBs is also required for the accumulation of BRCA1-BARD1 [48,54,70,83]. A fraction of BRCA1-BARD1 is recruited to DSBs via the BRCA-A complex that contains the BRCA1 interacting partner Abraxas and the dual SUMO2/3 and K63-Ub interacting factor RAP80 (UIMC1/ubiquitin interaction motif containing 1), which serves as a docking factor for the complex [86–88]. DNA damage-dependent SUMOylation of NPM1 (nucleophosmin) promotes interaction with the SIM motif in RAP80. In turn, this reduces BRCA1 interaction with CtIP, reducing DNA end-resection [89]. The SUMO-binding ZMYM2/3 proteins are recruited to DSBs through SUMO interactions where they promote BRCA1 recruitment to limit 53BP1 spreading [90,91].

## RPA (replication protein A 1/2/3 complex) and RAD51

**Figure 6.**
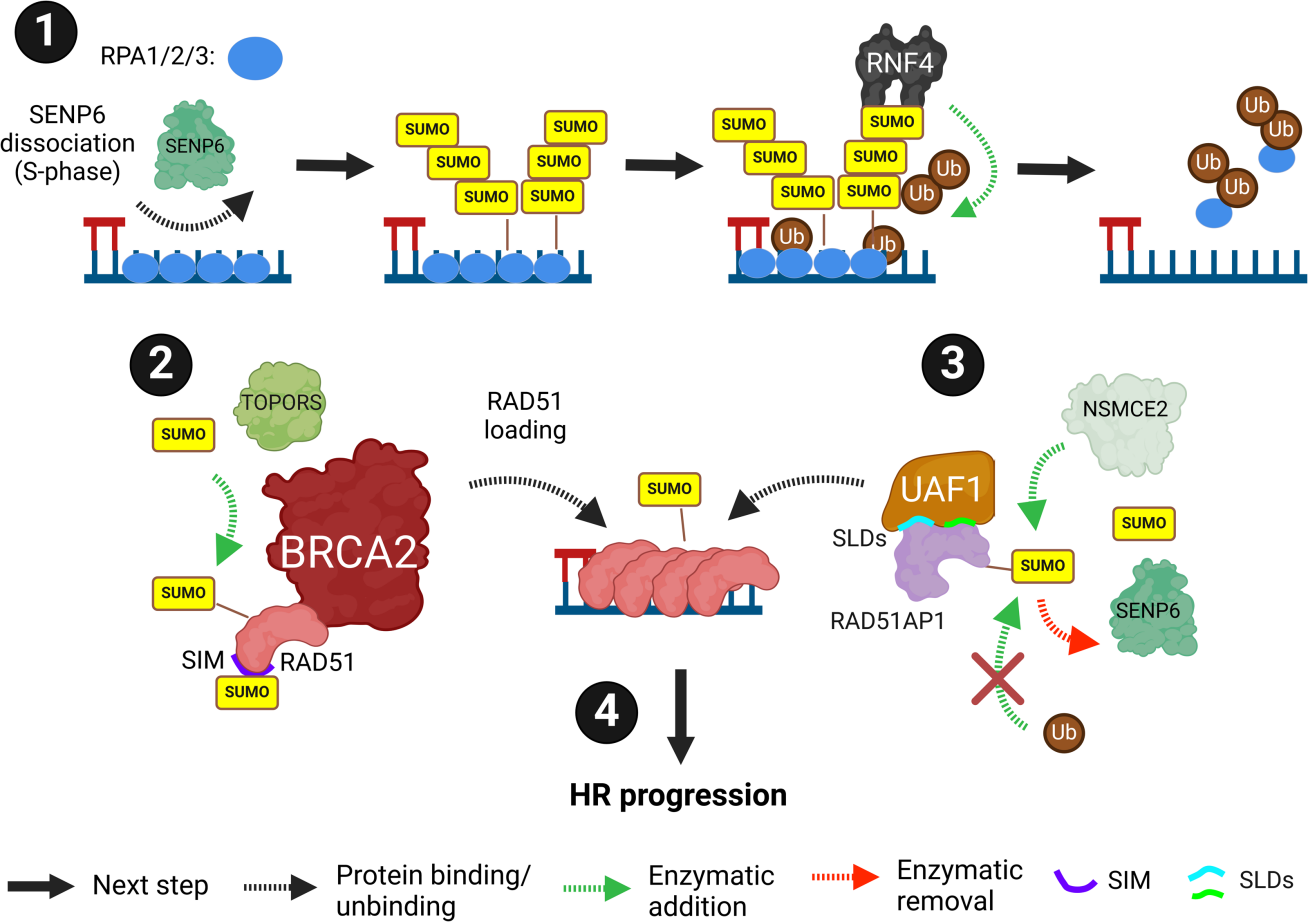
RPA (Replication Protein A 1/2/3 complex) and RAD51. SUMOylation is required for RPA1/2/3 dynamics and RAD51 filament formation. (**1**) RPA1/2/3 dissociates from SENP6 and binds the resected ssDNA. This dissociation promotes an increase in SUMOylation. SUMOylation of RPA1 is needed for RAD51 filament formation but can be removed via RNF4-dependent ubiquitination. (**2**) TOPORS SUMOylates RAD51 which promotes its interaction with BRCA2 and its subsequent loading to ssDNA. A SIM in RAD51 is also required for its HR function. (**3**) UAF1 binds RAD51AP1 SIMs via two SLDs. NSMCE2 SUMOylates RAD51AP1 which prevents its ubiquitination. SENP6 deSUMOylates RAD51AP1. (**4**) This aids in RAD51 filament formation, promoting the progression of HR to resolve DSBs. Created with BioRender.com.

DNA-end resected stretches of single-stranded DNA are coated with trimeric RPA complex (RPA1/2/3). RPA serves as an important signalling platform as well as protecting the ssDNA. During S-phase RPA1 dissociates from SENP6 promoting increased SUMOylation that is necessary for RAD51 filament formation and HR (Figure 6, subsection 1). RNF4 subsequently ubiquitinates RPA1 reducing its residency at resected DNA [67,92]. SUMOylated HNRNPA2/B1 (heterogeneous nuclear ribonucleoproteins A2/B1) limits HR repair by competing with ATRIP (ATR interacting protein) for RPA1 interaction. SENP1-dependent deSUMOylation of HNRNPA2/B1 reduces this interaction allowing the progression of HR [93]. The RAD51 loader BRCA2 facilitates RPA1-3 displacement from ssDNA.

TOPORS-dependent RAD51 SUMOylation promotes interaction with BRCA2 and loading [94] (Figure 6, subsection 2). RAD51 also interacts non-covalently with SUMOylated BLM, which promotes HR [95]. A RAD51 SIM is required for its accumulation at DSBs and HR repair [96]. The UAF1-RAD51AP1 (RAD51 associated protein 1) interaction is critical for the formation of the synaptic nucleoprotein intermediate required for RAD51's HR-promoting function. A small number of mammalian proteins contain SUMO-like domains (SLDs) which mimic SUMOs and can be recognised by SIMs. Two SLDs in UAF1 interact with a SIM in RAD51AP1 in a trimeric complex with RAD51 to aid the formation of the synaptic nucleoprotein complex [97,98]. RAD51AP1 is also SUMOylated [99,100] by NSMCE2 and deSUMOylated by SENP6. The SUMOylation of RAD51AP1 is competitive with ubiquitination and promotes stability [99] (Figure 6, subsection 3).

## Replication associated DSBs

DNA replication stress (RS) is a major source of endogenous DSBs due to the cleavage of stalled replication forks [101]. Many DNA repair proteins are SUMOylated under conditions of RS [56,102–105]. Signalling of the RS master kinase ATR (ataxia telangiectasia and Rad3-related protein) is aided by SUMO-dependent protein-protein interactions within the ATR-ATRIP complex and through SUMOylation-mediated stabilisation [60,93,106–109].

The chromatin surrounding the replisome is enriched in SUMOylation but depleted in ubiquitination by replisome-associated USP7 (ubiquitin-specific protease 7) deconjugating ubiquitin from SUMO conjugates [110,112]. CRISPR knockout screens in RNF4^−/−^ cells have also identified USP7 as a cooperative factor in maintaining ubiquitin pools essential for DNA replication [113]. RNF4 recruits to stalled replication forks via its SIMs and has roles in fork stability, collapse, and recovery during RS [110,113–117].

**Figure 7.**
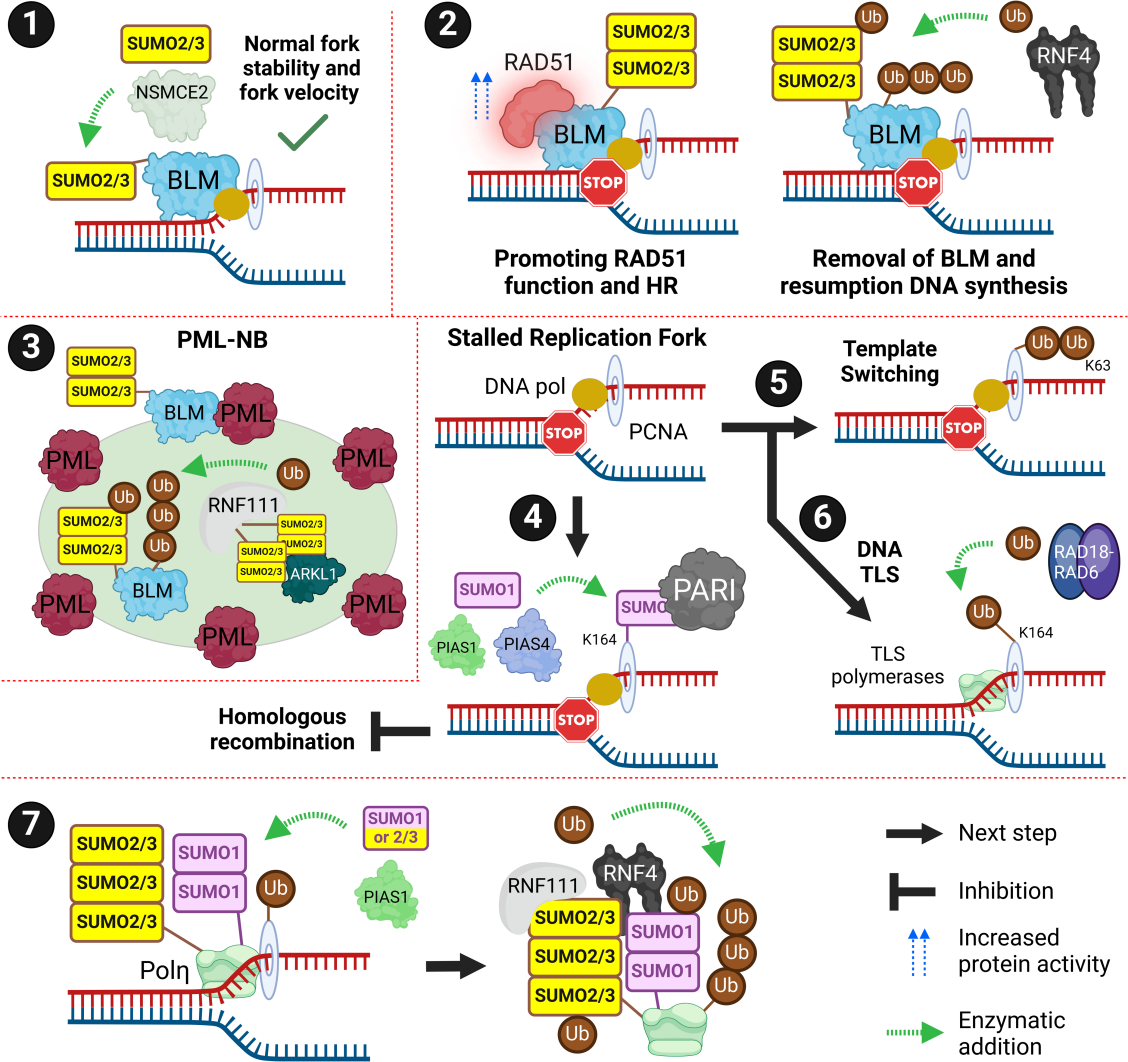
Replication associated DSBs. PCNA PTMs alter pathway choice. (**1**) SUMOylated BLM is required for optimal replication and helps stabilise the replication fork and maintain fork velocity. BLM is SUMO2/3ylated by NSMCE2. (**2**) BLM is also involved in repairing damaged replication forks. SUMOylated BLM can recruit and promote RAD51 function leading to repair via HR. Once DNA damage has been repaired, SUMOylated BLM is targeted for removal by RNF4-dependent ubiquitination to allow for the resumption of DNA replication. (**3**) BLM is associated with PML-NBs. SUMOylation of BLM promotes its association with PML in PML-NBs. It also permits the ubiquitin-dependent degradation of BLM via RNF111/ARKL1 which is essential for BLM turnover. RNF111 is SUMOylated at K237 and K238 which interacts with SIMs on ARKL1. (**4**) SUMO1ylation of PCNA at K164 by PIAS1/4 recruits PARI which inhibits repair via HR. (**5**) Template switching occurs when PCNA is polyubiquitinated with K63 chains. This involves HR with an undamaged sister chromatid. (**6**) Translesion synthesis (TLS) occurs when PCNA is monoubiquitinated at K164, providing a binding surface for TLS polymerases. (**7**) SUMOylation of TLS polymerase, Polη, by PIAS1 is required for the RNF4/RNF111-ubiquitin dependent removal of Polη. This ensures that Polη is removed when not required and replaced by replicative DNA polymerases to prevent mutagenic Polη activity. Created with BioRender.com.

The BLM (Bloom syndrome) helicase is SUMOylated by the NSMCE2 E3 component of the SMC5/6 complex which localises to stressed replication forks. RNF4 subsequently ubiquitinates SUMOylated BLM to aid in its removal which is required for the resumption of DNA synthesis. BLM helicase has multiple roles in DSB repair, DNA replication and resolving topological DNA structures such as G4 quadruplexes. BLM-SUMO interactions and BLM-SUMOylation also contribute to the promotion of RAD51 loading and HR repair at damaged replication forks. SUMOylation of BLM promotes its localisation to PML-NBs and interaction with PML itself [47,95,114,118–120]. BLM protein ubiquitination and turnover in PML-NBs is regulated by a SUMO-dependent association between RNF111 and its paralog ARKL1 (Arkadia-like 1). The RNF111-ARKL1-induced turnover of BLM reduces the resolution of G4 quadruplexes [121]. Collectively SUMO can therefore regulate BLM turnover at both replication forks and PML-NBs and can promote and impede BLM genome stability functions (Figure 7, subsections 1–3).

Topoisomerase-2 (TOP2) is recruited to stalled replication forks where it is SUMOylated by ZNF451. SUMOylation of TOP2 promotes recruitment of the DNA translocase Plk1-interacting checkpoint helicase via SIM interactions which aids in slowing and reversing the replication fork to preserve genome stability [122].

## DNA damage tolerance pathways

The DNA replication machinery stalls when it encounters lesions that have not been repaired and prolonged stalling results in replication fork collapse, generation of a DSB and genome instability. Template switching (TS) uses an undamaged sister chromatid as a temporary template for HR. DNA translesion synthesis (TLS) switches the precise and efficient replicative polymerases α/δ/ε with specialised low processivity error-prone TLS polymerases (ι/η/κ/ξ and Rev1) that can replicate across the damaged DNA [123].

The replication processivity factor PCNA (proliferating cell nuclear antigen) encircles DNA and travels with the DNA replication fork. On stalling, PCNA becomes monoubiquitinated at K164 by RAD18-RAD6 which provides a binding surface for TLS polymerases. Extension of the PCNA monoubiquitination into ubiquitin K63-linked polymers promotes TS [124]. PCNA is also SUMOylated at K164 by SUMO1 and SUMO2/3. SUMO1ylation of PCNA promotes the recruitment of the helicase PARI (PCNA-associated recombination inhibitor) which suppresses HR at replication forks [125–127]. In B cells PIAS1/4 PIAS1/4-dependent PCNA SUMO1ylation also promotes a shift from TS to TLS [128]. Distinct from SUMO1ylation, SUMO2/3ylation of PCNA by TRIM28 (tripartite motif containing 28) has roles in the resolution of transcription-replication conflicts [129]. Polη is a TLS polymerase SUMOylated after UVC damage. This SUMOylation triggers RNF4-RNF111-dependent extraction of Polη from sites of DNA damage to prevent mutagenic TLS [130]. SUMOylation therefore can both promote and limit mutagenic TLS (Figure 7, subsections 4–7).

## Inter-strand cross-link repair

**Figure 8.**
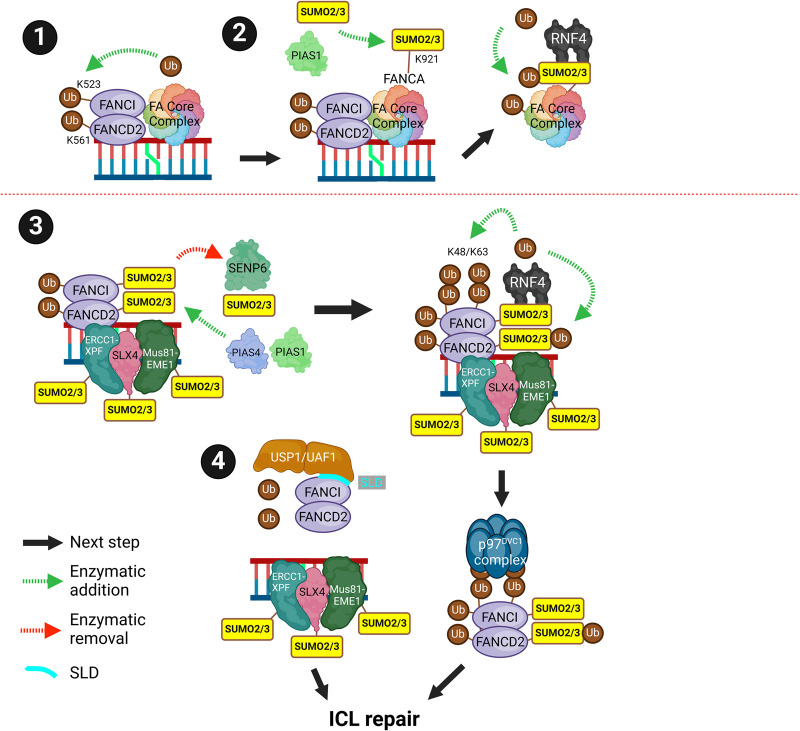
Inter-strand cross-link (ICL) repair. (**1**) The FA core complex recognises ICLs and ubiquitinates the ID2 complex at K523 and K561. Ubiquitination of the ID2 complex recruits ICL effector proteins (shown in (**3**)). (**2**) The FANCA subunit of the FA core complex is SUMOylated at K921 by PIAS1. SUMOylation of FANCA is required for the RNF4-depedent ubiquitination and clearance of the FA core complex. (**3**) The ID2 complex is SUMOylated by PIAS1 and 4 and deSUMOylated by SENP6. This leads to the RNF4-dependent ubiquitination of ID2 and extraction by VCP/p97. (**4**) SUMO-like domains (SLDs) in UAF1 interact with a SIM in FANCI to promote the recruitment of the USP1-UAF1 complex to ID2, promoting deubiquitination. ICL effector proteins (SLX4 and associated nucleases) are also SUMOylated. Created with BioRender.com.

The Fanconi anaemia (FA) core complex consists of multiple FANC proteins that recognise inter-strand cross-links (ICLs) and contains a ubiquitin E3 ligase that monoubiquitinates the heterodimeric ID2 complex (FANCI/FANCD2). Monoubiquitination of the ID2 complex recruits the ICL effector proteins. These effectors include nucleases, SLX4 (structure-specific endonuclease subunit 4), ERCC1-XPF and Mus81-EME1 which generate the nucleolytic incision flanking the ICL to unhook the cross-link. TLS is used to bypass the cross-linked nucleotides on the complementary strand. The incision results in a DSB which is repaired by HR, and NER fills in the gap. Finally, the monoubiquitinated ID2 complex is deubiquitinated by USP1-UAF1 (ubiquitin specific protease 1 — USP associated factor 1), which promotes its clearance [131] (Figure 8, subsections 1 and 2).

A mutation in FANCA (I939S) disrupts interaction with FAAP20 (FA core complex associated protein 20) within the FA core complex resulting in exposure of a SUMOylation site, once SUMOylated FANCA is degraded by RNF4 [132]. FANCI/D2 is SUMOylated by PIAS1/4 at ICLs, promoting RNF4-dependent ubiquitination and extraction by the VCP/p97^DVC1^ complex (Figure 8, subsection 3). This response is antagonised by SENP6 which deSUMOylates FANCI/D2 [107,133]. The SLDs of UAF1 interact with the SIMs of FANCI to promote the recruitment of USP1-UAF1 and de-ubiquitination of ID2 [134]. Therefore SIM-SLD interactions support the ubiquitin USP1-UAF1 dependent removal of ID2 and in parallel, SUMO conjugation and ubiquitination provide an alternative route of ID2 extraction from chromatin (Figure 8, subsection 4).

SLX4 (FANCP) and SLX1 act as scaffolds for nucleases required for the resolution of ICL and HR recombination intermediates [135]. SLX4 interacts with SUMO, is SUMOylated and may be a SUMO E3 ligase [44,52,107,136–140]. SUMO-SIM interactions by SLX4 help drive the formation of molecular condensates, compartmentalising the SUMO-RNF4 system to trigger selective modification of SUMO substrates [139]. The SUMO binding function of SLX4 is needed for the recognition of DNA damage sensors MRN, RPA and TRF2 (telomeric repeat binding factor 2) [137]. SLX4 SUMOylation is also promoted by NIP45 (nuclear factor of activated T cells 2 interacting protein), an SLD-containing protein that stimulates Ubc9 activity towards a subset of proteins [140,141]. SUMOylated SLX4 also facilitates the resolution of catenated DNA structures before M-phase [140]. The nucleases Mus81 and EME1 are SUMOylated which has important roles in genome stability [47,52,139,142] (Figure 8).

## SUMOylation and DNA-protein cross-link repair

**Figure 9.**
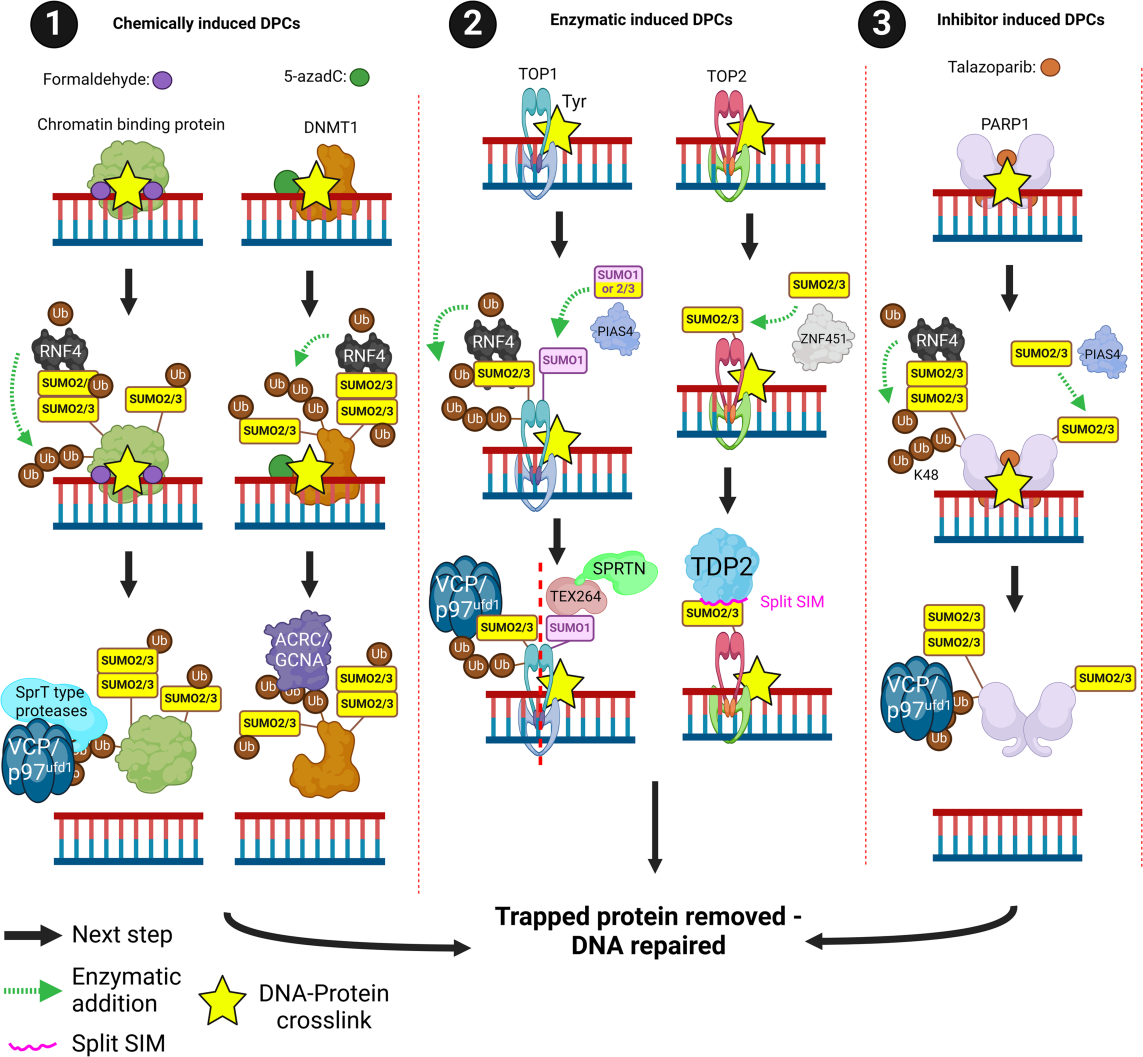
SUMOylation and DPC repair. DPCs can be induced by (**1**) chemical cross-links, (**2**) enzymatic trapping or (**3**) inhibitor trapping. (**1**) Chemically induced DPCs by formaldehyde can trap chromatin-binding proteins. These chromatin-binding proteins are SUMO2/3ylated and then RNF4-dependent ubiquitinated. SprT type proteases remove the trapped proteins in cooperation with the VCP/p97^ufd1^ complex. 5-azadC is a DNMT1 inhibitor that traps DNMT1 on DNA. SUMO2/3ylation of DNMT1 leads to RNF4 ubiquitination and subsequent removal by ACRC/GCNA proteases. (**2**) Resolution of enzymatic-induced TOP1 DPCs require the SUMO1,2/3ylation of TOP1. This leads to SPRTN-dependent clearance of TOP1 via TEX264 or VCP/p97^ufd1^ clearance of TOP1 via RNF4. Resolution of enzymatic-induced TOP2 DPCs require the SUMO2/3ylation of TOP2 by ZNF451. This leads to the removal of trapped TOP2 by TDP2 via a ‘split’ SIM that only forms in a 3-dimensional manner. (**3**) Inhibitor-induced PARP1 DPCs by Talazoparib requires SUMO2/3ylation for resolution. SUMO2/3ylation of PARP1 is conducted by PIAS4 which leads to RNF4-dependent ubiquitination. The VCP/p97^ufd1^ complex then removes the trapped PARP1. Created with BioRender.com.

DNA-protein cross-links (DPCs) are DNA-protein adducts that disrupt the normal functioning of DNA. They can be generated during enzymatic transactions on DNA (e.g. topoisomerases 1-2 Top1/Top2) [143] by chemicals that cause protein-DNA cross-links (e.g. aldehydes including formaldehyde) or by agents that trap enzymes on DNA (camptothecin (Top1), etoposide (Top2) and some PARP1/2 inhibitors). Formaldehyde and the DNMT1 (DNA methyltransferase 1) inhibitor 5-azadC (5-aza-2′-deoxycytidine) promotes global increases in chromatin SUMOylation, in the case of 5-azadC, DNMT1 is the major SUMOylated substrate [144–146]. These SUMOylated DPCs are cleared by a combination of RNF4-dependent ubiquitination and SprT-type proteases SPRTN and ACRC/GCNA (germ cell nuclear acidic peptidase) [144,146] (Figure 9, subsection 1).

TOP1 and TOP2-DPCs are also degraded in a SUMO-ubiquitin-dependent manner. TOP1-DPCs are sequentially modified by SUMO2/3, SUMO1 and ubiquitin using PIAS4 and RNF4 [147]. VCP^SPRTN^-associated TEX264 (testis expressed 264, ER-phagy receptor) interacts with SUMO1-modified TOP1 promoting TOP1-DPC clearance [148]. The SUMO E3 ZNF451 SUMOylates TOP2-DPCs and promotes the recruitment of TDP2 (tyrosyl-DNA-phosphodiesterase) via a ‘split’ or confirmational SIM motif. TDP2 enzymatically clears the TOP2-DPC adduct from DNA. ZNF451 therefore has a substantial impact on sensitivity to the TOP2 poison etoposide [122,149–151]. In addition to the SUMO-Ubiquitin dependent clearance of TOP2-DPC and the TDP2 enzymatic removal pathway, SUMOylation of TOP2 and ZNF451 increases their interaction with the ATPase RAD54L2 which can extract the TOP2-DPC from chromatin [151,152]. The SUMO-ubiquitin proteasome-mediated TOP2-DPC clearance pathway promotes DSB formation and sensitivity to etoposide, ultimately leading to genomic instability, suggesting this is not a preferred repair pathway [151,153] (Figure 9, subsection 2).

The PARP inhibitor Talazoparib causes PARP1 ‘trapping’, locking it onto DNA forming DPC-like adducts. SUMOylation of PARP1 by PIAS4 promotes RNF4-dependent ubiquitination followed by VCP/p97^Ufd1^-mediated clearance [154] (Figure 9, subsection 3).

## SUMOylation and chromatin

The chromatin landscape plays a significant role in DDR [155,156]. The SUMO proteome is enriched in chromatin remodellers which are dynamically modified during DNA damage signalling [11,146,157]. SUMOylation regulates the eviction and deposition of histones, including H2AZ during DDR [158,159]. DNA damage-induced chromatin relaxation which is essential for repair factors access to DNA is aided by SENP7-dependent deSUMOylation of TRIM28, which attenuates its SIM-dependent interaction with the NuRD^CHD3^ remodelling complex [73]. SENP1 also promotes chromatin relaxation by countering TRIM28-dependent SUMOylation of MORC2 during the early DSB response [160].

## Perspectives

- SUMOylation is prevalent in most DNA repair pathways. Deficiencies in SUMO signalling disrupt and reduce DNA repair proficiency and increase sensitivity to chemotherapies used in cancer treatment. Several DDR pathways, both recently described (ribonucleotide excision repair) and established (mismatch repair) have not yet been characterised for their reliance on SUMO.
- DDR signalling serves as an excellent example of SUMO group modification and de-modification models [44,47,107]. Examples where individual SUMO sites or substrates have substantive impacts are however also prevalent in DDR [66,83]. All the known SUMO signalling modalities, (modulation of protein-protein interactions, competition/cooperation with ubiquitin, and promotion of phase separation) are represented in DDR.
- Synthetic lethality in specific cancer types and DDR pathways can be exploited with SUMOylation inhibitors [145,161,162], in the future inhibitors of deSUMOylation could also have uses in DDR defect exploitations.

## Open Access

Open access for this article was enabled by the participation of University of Leeds in an all-inclusive *Read & Publish* agreement with Portland Press and the Biochemical Society under a transformative agreement with JISC.

## Abbreviations

ATM: ataxia telangiectasia mutated
BER: base excision repair
CPD: cyclobutane pyrimidine dimer
DPC: DNA-protein cross-link
DDR: DNA damage repair
DSB: double-strand break
FA: Fanconi anaemia
HR: homologous recombination
ICL: inter-strand cross-link
NER: nucleotide excision repair
NHEJ: non-homologous end joining
PCNA: proliferating cell nuclear antigen
PML: promyelocytic leukaemia
RS: replication stress
SBD: SUMO binding domain
SLD: SUMO-like domain
SSBR: single-strand break repair
TDG: thymine DNA glycosylase
TDP1: tyrosyl DNA phosphodiesterase 1
TLS: translesion synthesis
TS: template switching
